# Histological quantification of decomposed human livers: a potential aid for estimation of the post-mortem interval?

**DOI:** 10.1007/s00414-020-02467-x

**Published:** 2020-11-24

**Authors:** Ann-Sofie Ceciliason, M. Gunnar Andersson, Sofia Nyberg, Håkan Sandler

**Affiliations:** 1 Forensic Medicine, Department of Surgical Sciences, Uppsala University Hospital, Uppsala University, SE-751 85 Uppsala, Sweden; 2 Department of Chemistry, Environment and Feed Hygiene, The National Veterinary Institute, SE-751 89 Uppsala, Sweden; 3 Department of Forensic Medicine, The National Board of Forensic Medicine, Box 1024, SE-751 40 Uppsala, Sweden

**Keywords:** Forensic taphonomy, Post-mortem interval estimation, Hepatic decomposition score, Total body score

## Abstract

The objective of this study was to determine if a novel scoring-based model for histological quantification of decomposed human livers could improve the precision of post-mortem interval (PMI) estimation for bodies from an indoor setting. The *hepatic decomposition score* (HDS) system created consists of five liver scores (HDS markers): cell nuclei and cell structure of hepatocytes, bile ducts, portal triad, and architecture. A total of 236 forensic autopsy cases were divided into a training dataset (*n* = 158) and a validation dataset (*n* = 78). All cases were also scored using the *total body score* (TBS) method. We specified a stochastic relationship between the log-transformed accumulated degree-days (log_10_ADD) and the taphonomic findings, using a multivariate regression model to compute the likelihood function. Three models were applied, based on (i) five HDS markers, (ii) three partial body scores (head, trunk, limbs), or (iii) a combination of the two. The predicted log_10_ADD was compared with the true log_10_ADD for each case. The fitted models performed equally well in the training dataset and the validation dataset. The model comprising both scoring methods had somewhat better precision than either method separately. Our results indicated that the HDS system was statistically robust. Combining the HDS markers with the partial body scores resulted in a better representation of the decomposition process and might improve PMI estimation of decomposed human remains.

**Supplementary Information:**

The online version contains supplementary material available at 10.1007/s00414-020-02467-x.

## Introduction

Estimation of the post-mortem interval (PMI) is one of the most complicated tasks in forensic practice. A more reliable PMI estimate would be of great value in forensic investigations, where it could for instance help to elucidate the course of events, as well as to evaluate suspects’ alibis based on their whereabouts during the timespan of a suspected crime. Consequently, there is a constant desire to identify good predictors of the PMI that would yield a more accurate estimate or at least assign a narrower time interval. In decomposed human remains, the PMI is especially difficult to estimate with certainty due to the complexity of the decomposition process. The indoor decomposition of human remains has not been studied to the same extent as that in various outdoor environments [1]. The majority of decomposed human remains are found in an indoor setting [1, 2], highlighting the importance to further investigate this specific environment.

The most decisive factor affecting the rate of decomposition is the ambient temperature; a higher temperature speeds up the onset of post-mortem tissue changes and bacterial growth as well as enzymatic function [3, 4]. An increase in the decomposition rate can be seen when the body is covered with clothes, which may slow the natural cooling of the body, and if a longer time elapses between death and artificial cooling, i.e. transfer to a morgue facility [3, 5]. Other extrinsic factors of importance include ventilation and humidity; dry areas with a constant air flow cause rapid dehydration of a dead body, reducing bacterial growth and inducing mummification, as opposed to humid climates, which accelerate decomposition [3, 5].

Intrinsic factors affecting the decomposition process include obesity, the presence of open wounds, ante-mortem intoxication, infectious disease, and fever. Subcutaneous and abdominal fat acts as insulation, slowing the course of natural cooling [3–5]. Open wounds offer an entry point for bacteria and fungi, whereas ante-mortem infections imply an already increased bacterial load [5, 6]. Conversely, exposure to antibiotics or a large blood loss shortly before death may slow bacterial growth and hence the rate of decomposition [5]. As for ante-mortem intoxication, an overdose of, for example, paracetamol would cause the onset of liver necrosis and degradation before death [7]. Ante-mortem treatment of a disease may also alter the decomposition rate [2].

In an indoor setting, a dead body may be more protected from external factors than in an outdoor environment, as the body is not exposed to rain, wind, large fluctuations in temperature, or solar radiation. Animal scavenging and insect access are often limited in an indoor setting. It is therefore possible that other factors, such as body size, clothing, body position at death, pre-existing disease, and pathological changes, may have a larger impact on how the decomposition progresses. Quantification of decomposition based on visual changes to the body’s exterior, for example, the total body score (TBS) method [8], only captures a part of the decomposition process. The decomposition of the internal organs would be of interest to evaluate in order to get a better representation of the complete decomposition process that has taken place under specific circumstances (e.g. in an indoor setting).

The largest burden of bacteria is that in the ileocecal area. After death, these bacteria spread to the liver and spleen, and then to the heart and brain, creating a possible domino effect in the order that different organs and tissues decompose [9]. The liver is an organ with a well-defined histology and is well-protected from, for example, infestation by maggots thanks to its location in the abdomen. Furthermore, the liver has the highest quantity of post-mortem bacterial taxa of all internal organs, probably due to its central role in all metabolic processes, as well as its placement in close proximity to the gastrointestinal tract. It is also easily accessible for pancreatic enzymes and gallbladder fluids, potentially resulting in rapid autolysis [9]. Only a few previous studies of human livers have been carried out evaluating post-mortem histological changes and their potential association with the PMI [10–12]. However, these studies have focused on the first few days after death. To date, there are several well-established methods for PMI estimation of the early PMI period [13]. More extended PMIs have not been investigated to the same degree, and more reliable methods are still needed.

The overall objective of this study was to determine if a novel scoring-based model for histological quantification of decomposed human livers could improve the precision of PMI estimation. The first aim was to define and quantify PMI-dependent decomposition changes in the human liver. The second was to construct a scoring-based model and test its reliability and validity. The third aim was to further investigate use of this liver scoring-based system in combination with the total body score (TBS) method, applied to bodies found in an indoor setting.

## Materials and methods

### Selection of cases

In this study, a total of 236 forensic autopsy cases at the Department of Forensic Medicine in Uppsala, Sweden, were scored for external decomposition changes in accordance with the TBS system [8]. Liver histology slides from each case were also evaluated. The inclusion criteria were as follows: body found indoors (e.g. in an apartment or house), the deceased being of adult age (> 18 years), and the time of death being known. Furthermore, bodies that had been burned, submerged (i.e. in a bathtub), or suffered severe injuries from trauma or carnivore scavenging were not included due to possible altered decomposition rate and/or patterns [6, 14–16]. Cases with prominent liver pathology were not included. Pathological changes, such as severe steatosis and/or cirrhosis, greatly altered the liver architecture giving these cases a different appearance [17]. Staging of fibrosis/cirrhosis was done according to the Metavir score. Pathological liver samples of score F1 to F3 were accepted, but not F4 (i.e. cirrhosis with bridging fibrosis and nodular regeneration). Steatosis grades I and II were accepted but not III (i.e. severe, with more than 66% fat deposition in hepatocytes). The female to male ratio was 1:4 and the median age at death was 64.5 years (range 21 to 96 years). Body mass index (BMI) was from 11.2 to 53.1 kg/m^2^ (median: 23 kg/m^2^).

The original dataset (dataset 1) was collected during 2010 to 2015 and consisted of 82 cases, all of which were used in the construction of the scoring-based hepatic decomposition system (and also re-scored with the finalised model). A new dataset (dataset 2) was collected during 2016 to 2018 and consisted of 154 cases. They were scored using the finalised version of the liver scoring model that had been constructed. Due to a dominance of summer cases in dataset 1 (*n* = 82), the new cases in dataset 2 were selected with a dominance of winter cases, in order to achieve a more even seasonal distribution in the complete dataset (see Fig. S1 in supplement).

### The total body score method

**Fig. 1:**
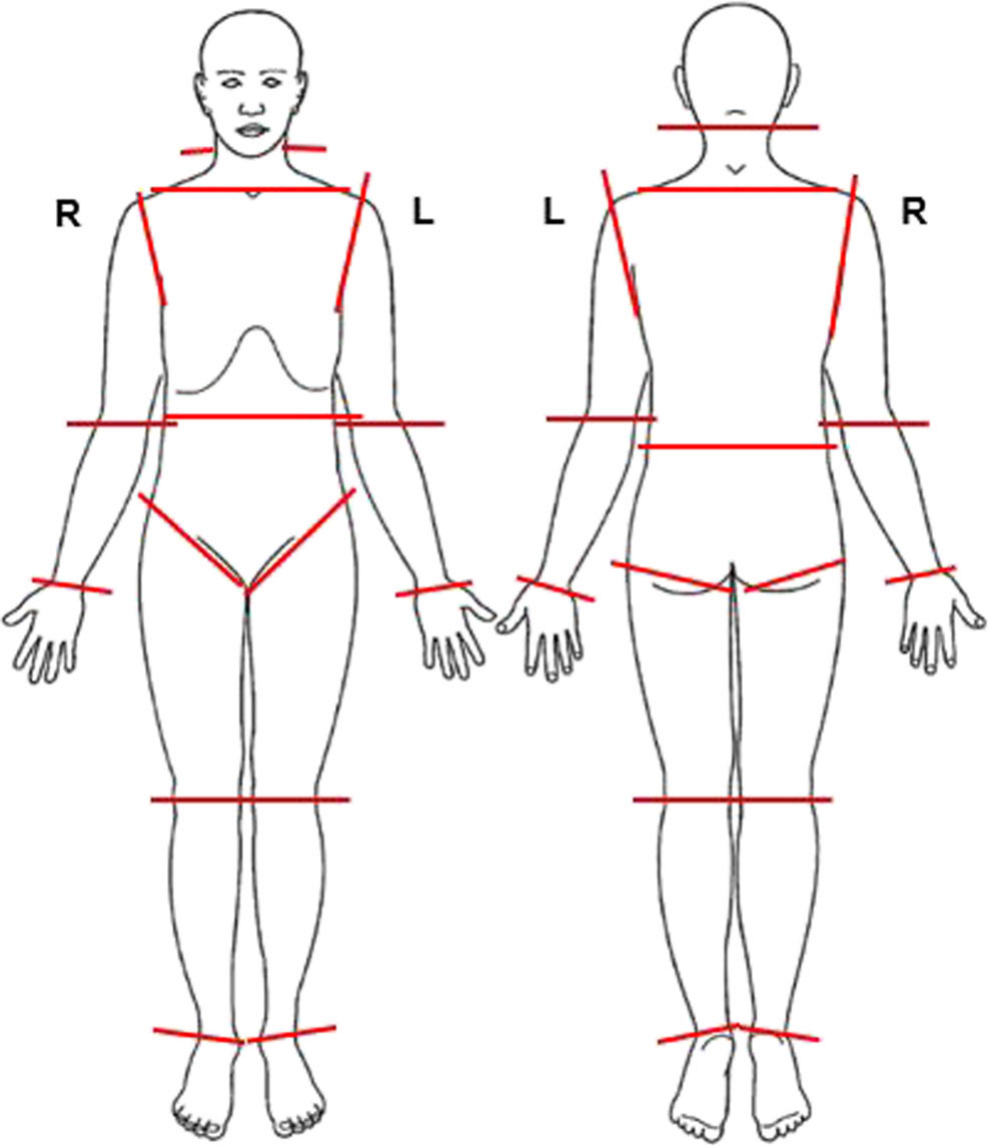
Body chart used for assigning scores during autopsy

The external post-mortem changes were assessed using the TBS method described by Megyesi et al. [8], who incorporated a scoring feature to enable a quantitative measure of decomposition. The original descriptions of each stage were applied in our study. In our model setup, non-decomposed or “fresh” remains corresponded to TBS of 0 [18], i.e. the scale started at 0 (zero), not 1 (one) as in Megyesi’s original study [8]. Three partial body scores—the head and neck (PBSH), limbs (PBSL), and the trunk (PBST)—were calculated. In Megyesi’s original study, the partial body scores were added up, resulting in a *total body score* (TBS). In this work, we slightly modified the TBS model and used a body chart protocol encompassing both front and back views of the body (Fig. 1). We divided the body into 32 anatomical regions, each scored independently at the autopsy. The three partial body scores were calculated from the local scores of these 32 anatomical regions (i.e. PBSH 4 regions, PBSL 24 regions, and PBST 4 regions). When the decomposition was unevenly distributed, an average was calculated, i.e. we considered the possible differences between, for example, the ventral and dorsal parts of the body. For example, PBST was calculated in the following way: 4 (upper torso) + 4 (abdomen) + 2 (upper back) + 2 (lower back) = 12/4 = 3. The individual partial body scores (PBSH, PBSL, and PBST) were later used in the statistical model.

### Presence of insect activity and external desiccation

In addition to the partial body scores, we noted insect activity, e.g. presence of nymphs, larvae, maggots, and/or flies, on the body chart protocol. The insect activity within a specific anatomical region was not quantified. Desiccation was also noted as presence of parchment- or leather-like desiccation of the skin within one or more anatomical regions. Local desiccation of fingers and/or toes did not result in labelling the case as desiccated.

### The post-mortem interval

The known time of death was estimated based on witness statements of when the deceased was last seen alive, or was last known to be alive according to circumstantial evidence stated in the police report (e.g. date of the most recent newspaper left in the mailbox, prescriptions, telephone calls, expiration dates on milk in the refrigerator). The time elapsed between last seen or known to be alive and discovery of the dead body was used as the PMI in our study, yielding a possible overestimation. Short PMIs would be prone to larger relative errors than long PMIs.

### The ambient indoor temperature and accumulated degree-days

The indoor temperature is normally well-regulated in Sweden and in the range of + 18 °C to 23 °C, with a typical temperature of between + 21 °C and 22 °C [19]. After discovery, the dead bodies were stored in a morgue facility at a temperature of + 5 °C ± 1 °C until the time of autopsy. The majority of cases underwent autopsy after 2 to 6 days storage (median = 4 days). In our model setup, morgue time represents the time interval between discovery of the dead body and the autopsy. This includes the transport time of the dead body to the morgue. In accordance with the conventions of the metric system, *accumulated degree-days* (ADD) is the unit for the *accumulated temperature* (AT). For the sake of simplicity, we use the term ADD instead of AT in this study. The ADD is the number of degrees above zero multiplied by the number of days passed since death (i.e. PMI + morgue time, see Eq. 1). Usually, the ADD would be calculated as an average of minimum and maximum temperatures during a day. However, in our setup, we only had one temperature indication, on the date of discovery of the dead body.

1 $$ \mathrm{ADD}=\mathrm{PMI}\ast {T}_{\mathrm{site}}+\mathrm{MI}\ast {T}_{\mathrm{morgue}} $$

MI is the morgue time, *T*_site_ is the temperature when and where the body was found, and *T*_morgue_ is the morgue temperature. Further, we used the log_10_ADD in the statistical analyses, in accordance with Megyesi et al. [8].

### Liver tissue processing

Several organs (e.g. heart, lung, liver, kidney, and pancreas) were routinely sampled during the forensic autopsies, placed in 10% formalin solution for fixation, and processed after 24- to 48-h fixation. Tissue preparation and later histological staining were performed by professionals at our department. The staining agents applied were *haematoxylin and eosin* (*HE*), for a distinct visual separation of cell nuclei and cytoplasm, and *Picro-Mallory trichrome* (*PM*), to show collagen in strong contrast with other structures. Liver tissue was not sampled specifically for this study. Instead, we retrospectively examined the liver slides available.

### Development of the hepatic scoring method

**Fig. 2:**
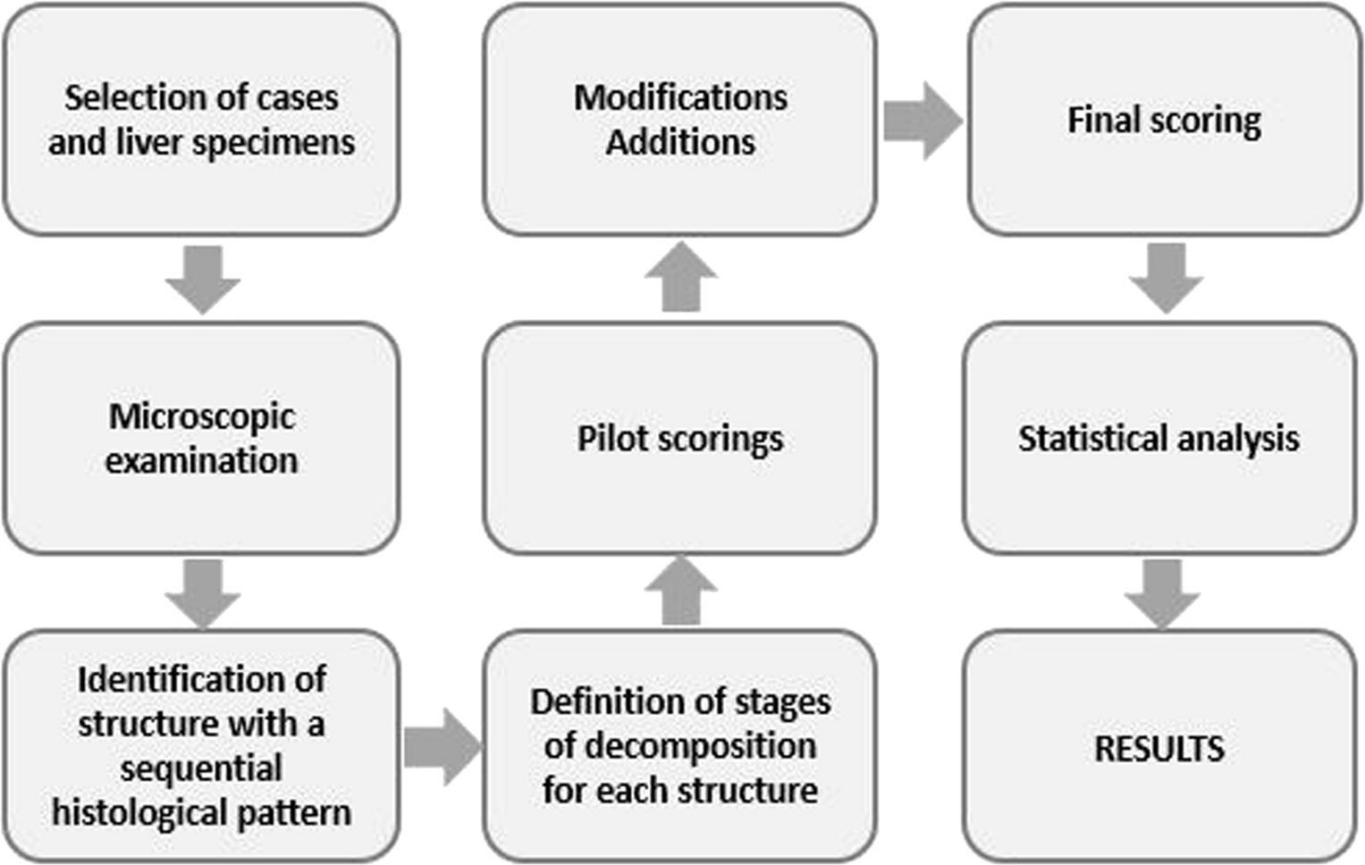
Method development

A schematic presentation of the method development is seen in Fig. 2. The aim of the initial light microscope examination of the 82 liver samples (dataset 1) was to identify histological changes in the tissue and cell structures that could be associated with the PMI, as well as to investigate the possibility of providing a tool for describing the degree of decomposition. At this initial examination, the different histological changes observed were documented (e.g. the degradation of hepatocytes, stroma, and portal areas). Presence of bacterial colonies and/or signs of gas formation (i.e. empty spaces within the liver tissue) were also noted. A suggested timeline of the observed decomposition changes was created and used as a foundation for the *hepatic decomposition score* (HDS). Histological changes appearing to be independent of the PMI were discarded during this process.

To construct a method feasible for a structured and systematic evaluation, as well as quantification of the extent of decomposition in the liver, a scoring-based system was created. This system encompassed the tissue structures and cell components, graded based on how much their microscopic appearance deviated from that observed in a fresh, unaffected sample (e.g. a liver biopsy), with special attention paid to definition and/or quantity.

The liver samples were then re-examined to facilitate the definition of observable stages of decomposition for each identified structure, ranging from optically maintained to non-identifiable. Each stage described was then translated into a score, where a low score indicated a low degree of decomposition, and a high score indicated a high degree of decomposition.

Several pilot scorings of the 82 specimens (dataset 1) were conducted with the objective of optimising the method. Difficulties in the interpretation of the written definitions and any possible inconsistencies concerning the identified structures or the scoring were noted, leading to additions or modifications of the system. The finalised version was later adopted for final scoring (all 82 liver samples were re-scored and these HDS results were used in the statistical analysis). To calibrate the scoring method, the authors had regular discussions about each case during the development process, to identify any contradictions in the scores given. These discussions indicated a need for a catalogue of photographs illustrating each score in the HDS system, as an aid in the scoring procedure.

## Statistical analysis

Microsoft Excel 2016 was used for general database handling. The statistical analysis was performed in R (https://www.r-project.org/).

### Testing the reliability of the HDS system

The inter-observer reliability was investigated at three separate occasions. The first inter-observer test was carried out during the method development (pilot scoring). Three observers (ASC, SN, HS) individually examined and graded 31 of the 82 liver samples (dataset 1). The second inter-observer test (final scoring) was carried out on an independent sample (40 cases randomly selected from the dataset 2, *n* = 154), not previously examined, blinded for the two observers (ASC, HS). To further examine the reliability of the HDS, a third inter-observer test was carried out with three independent observers with competence to evaluate histological slides but without prior knowledge of the HDS system, using an independent sample of 40 cases (randomly selected from the dataset 2, *n* = 154). The independent observers had a catalogue of photographs for each score (31 of the 82 cases used to create the HDS system) as help during the scoring, in addition to the HDS descriptions. They were also given the opportunity to test score the photographed cases and compare their results with those of one of the authors (ASC) before they scored the new samples. They did not have any discussions between themselves or with the authors concerning scoring cases. They scored each case individually, without knowledge of the scores given by the other observers.

Data analysis used intra-class correlation (ICC) and standard errors of measurement (SEM). The ICC is usually used as a measure of association when studying the reliability of observers and reveals similarities between test methods that measure a continuous variable (interval or ratio scale) [20]. SEM is considered preferable to other measures of observer variability since it is not sensitive to data range, which ICC is [21]. Calculations of ICC and SEM were carried out as presented by Popović and Thomas [21].

### Training data and validation data

Of the 236 cases, 2/3 were selected as training data for the statistical model and the remaining 1/3 as validation data. The original 82 cases (dataset 1) used in the development of the HDS system were included in the training data. Another 76 cases from dataset 2 were randomly selected based on month of discovery of the body (seasonal distribution). The two datasets, training (*n* = 158) and validation (*n* = 78), were made equal in regard to seasonal distribution (Fig. S1, supplement). The validation dataset encompassed dataset 2 cases only.

### Transformation of the HDS

Megyesi et al. [8] and Moffatt et al. [18] carried out power transformation of data in order to achieve a normal distribution and a linear relationship between the decomposition score (i.e. TBS) and the ADD. In our study, we applied the Box-Cox transformation to improve the fit of the model [18].

### Multivariate regression model

We established a stochastic model for our variables of interest. The model was then used to compute a likelihood function in cases where forensic evidence, i.e. our taphonomic data (HDS markers and/or partial body scores), was observed, but the PMI was unknown. The maximum likelihood (ML) method was used for training the model. This method is a way to determine the value of one or more parameters in a model that maximises the likelihood function, i.e. to find the values of these parameters that best explain the observed data.

We used the same statistical model as in our previous study [22]. To recapitulate, we applied the multivariate normal regression model:

2 $$ y\mid \theta \sim {\mathrm{Normal}}_3\left( a\theta +b,\Sigma \right) $$

where *θ* is the log-transformed accumulated degree-days log_10_ADD and *y* is the vector of the partial body score and/or the HDS markers. By using this model, we could capture possible residual covariance (i.e. how the partial body scores or HDS markers differed from the expected values). A training dataset with known PMIs and partial body scores was then used to obtain estimates for the model’s parameters, based on the following equation:

3 $$ \pi \left(y\mid \theta \right)\propto \mathrm{Normal}\left(\theta; {\left({\hat{a}}^t{\hat{\Sigma}}^{-1}\hat{a}\right)}^{-1}{\hat{a}}^t{\hat{\Sigma}}^{-1}\left(y-\hat{b}\right),{\left({\hat{a}}^t{\hat{\Sigma}}^{-1}\hat{a}\right)}^{-1}\right) $$

We could, for a new observed *y*, compute the likelihood.

In the current work, we used three models, consisting of (i) five HDS markers, (ii) three partial body scores, and (iii) HDS markers in combination with partial body scores (a total of eight scores). The multivariate regression model allowed PMI estimation even when partial body scores were missing [22]. We therefore tested masking one or more of the HDS markers and/or partial body scores, to investigate the performance of the statistical model. Further, we compared the predicted log_10_ADD with the true log_10_ADD of each case and applied the fitted model to the validation dataset, to determine if the model worked on an independent material.

## Results

In this section, we will begin by presenting the decomposition changes observed in the human liver in an indoor setting, and the hepatic decomposition score (HDS) system constructed. Next, we will proceed to the statistical analysis of the HDS system. Then, we will demonstrate the reliability and how we tested the model for validity in an independent dataset. We will also show data on outliers and the overall performance of the fitted model.

### The decomposition process in the liver

The general changes observed throughout the trajectory of decomposition in the liver are summarised in Table 1*.* The majority of cases with a PMI of less than 1 day are without visible changes (i.e. not detectable under a light microscope). A few cases with a PMI of 1 to 4 days also lacked visible changes. On the other hand, one case with a PMI of 3 days displayed an advanced state of decomposition, usually seen in cases with a PMI of 20 or more days. The end stage in this study was when only collagen remained and the tissue could no longer be identified as liver. This stage was usually reached at around 35 days after death. However, some cases with a PMI of 43 to 217 days (*n* = 12) had a slightly less decomposed tissue, still identifiable as liver. The common factor for these cases was widespread external desiccation.

**Table 1:** The general changes observed on the trajectory of decomposition in human livers (PMI up to 217 days) in an indoor setting. The results are based on examinations of conventional histological staining (HE, PM) and a magnification of × 100, × 200, and/or × 400

PMI (days)	Hepatocytes	Portal areas	Architecture
0–5	No visible changes, or darkened, dense cell nuclei. Sinusoids with intact red blood cells or signs of cell debris in the sinusoids.	Distinct or faded nuclei of bile duct epithelial cells. Portal triad distinct or mild scattering of collagen visible.	Typical hexagonal shape of lobules. Hepatocytes start to detach from each other.
6–10	Darkened, dense cell nuclei or shadow nuclei. Visible cell debris between the hepatocytes.	Faded nuclei of the bile duct epithelial cells or only cell residues left. The portal triad (artery, vein, and bile duct) still identifiable, with scattering of collagen.	Hexagonal shape of lobules may still be visible. The detachment of hepatocyte from the hepatic cords is evident.
11–20	Shadow nuclei or only a few nuclei still visible. Increasing amount of cell debris and some loss of cell material.	Mainly cell residues, still identifiable as bile ducts, blood vessels visible in the portal triad, fragmentation and scattering of collagen.	Lobules and sinusoids not visible. Detached hepatocytes scattered in a surrounding of cell debris.
21–30	A few shadow nuclei or nuclei not identifiable (i.e. total loss of cell nuclei). Some remnants of hepatocytes and cell debris remain, evident loss of cell material.	Bile ducts only as cell residues or not identifiable, some blood vessels still visible, fragmentation and scattering of collagen more evident.	Distorted hepatic pattern with scattered remnants of hepatocytes.
≥ 31	Cell nuclei not visible, complete disintegration of the hepatocytes with apparent loss of cell material, some cell debris may remain, in some cases only collagen left.	Bile ducts and blood vessels not possible to identify. Fragmentation and scattering of collagen or portal triad not possible to identify.	The hepatic pattern is extensively distorted or completely lost (i.e. tissue not identifiable as liver).

The general architecture of the liver changed progressively with increasing PMI (Table 1). The hexagonal pattern, representing hepatic lobules, was seen to gradually disappear, and the alignment of hepatocytes in cords was lost. The morphology of sinusoids (small blood vessels running between the hepatocytes) disintegrated progressively and was not visible after a PMI of > 5 days. Within the sinusoids, it was possible to find intact red blood cells in case of short PMIs (≤ 2–3 days). Early signs of cell debris in the sinusoids mainly consisted of red blood cells. Later, cell debris from the disintegrating hepatocytes was evident between the detached hepatocytes and the structure of sinusoids was no longer distinguishable. Lack of cell material was seen for longer PMIs and was often prominent after a PMI of 20 days.

**Fig. 3:**
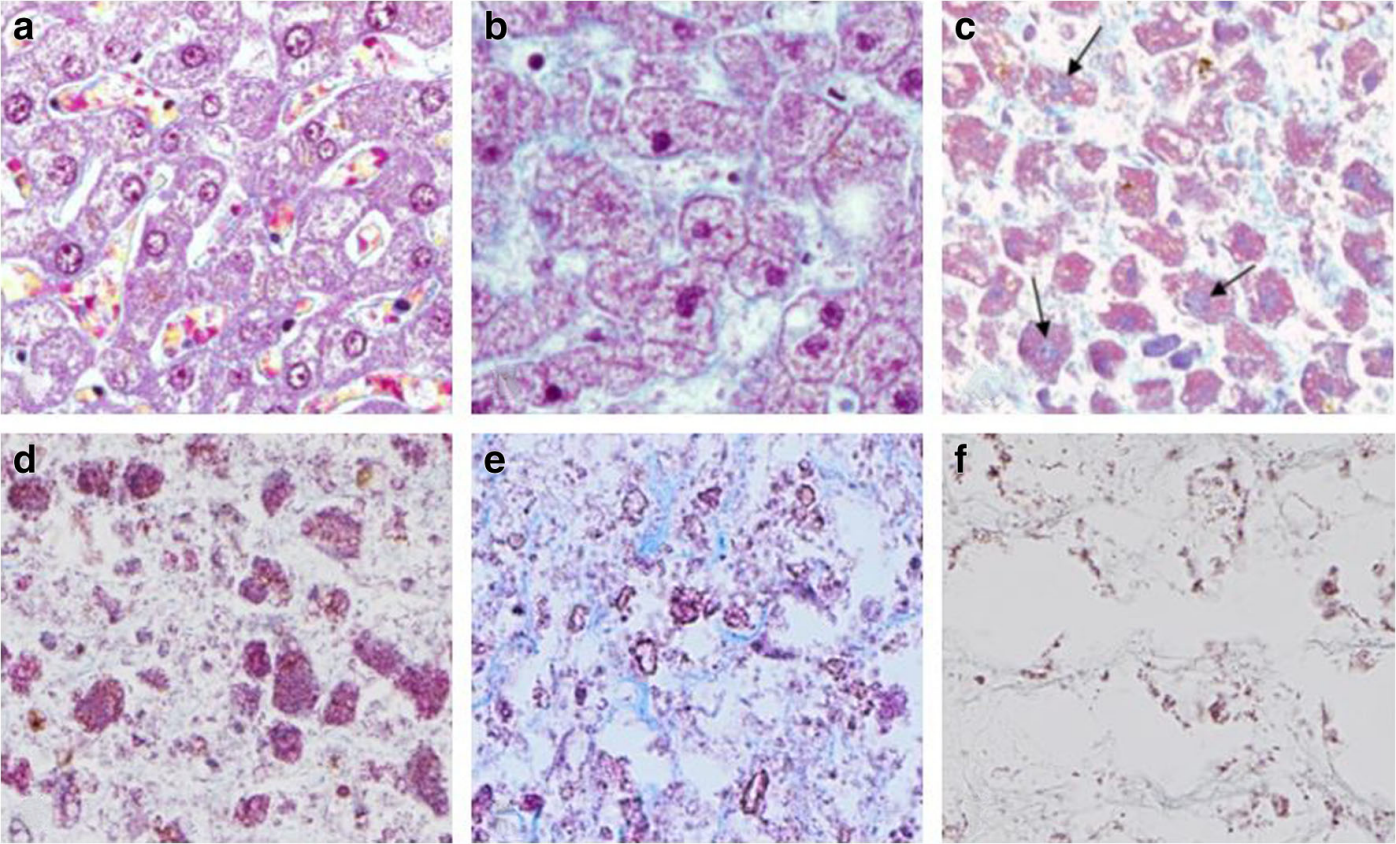
Decomposition of hepatocytes. PM (Picro-Mallory trichrome) × 400. **a** Distinct cell nuclei (*score 0*) and maintained cell structure (*score 0*). The sinusoids are also distinct, with intact red blood cells and alignment of hepatocytes. **b** Early signs of decomposition with darker and denser cell nuclei (*score 1*) and some cell debris in the sinusoids (*cell structure score 1*)*.* The hepatocytes are starting to detach from the hepatic cords. **c** Moderate disintegration of the hepatocytes with visible cell debris (*cell structure score 2*). The nuclei are seen as bluish shadows in the majority of the hepatocytes (black arrows) (*cell nuclei score 2*). The hepatocytes are beginning to appear more scattered and surrounded by cell debris and distorted collagen threads (light blue in colour). **d** Extensive disintegration of the hepatocytes, with cell debris and loss of cell material (*cell structure score 3*). The hepatic pattern is difficult to distinguish. A few bluish cell nuclei are visible in some remnants of hepatocytes (*cell nuclei score 3*). **e** Complete disintegration with some collagen remains (light blue) and cell debris (*cell structure score 4*). Cell nuclei not identifiable with certainty (*cell nuclei score 4*). **f** Not identifiable as liver tissue. Scattered and fragmented collagen (almost colourless or slightly light blue) and some brownish deposits or possible cell debris (*cell structure score 5*)

The cell nuclei of the hepatocytes have a particular progression of decomposition, going from distinct to indistinct and becoming darker in colour, finally becoming shadow nuclei. These shadow nuclei were not always visible in HE-stained samples. However, PM stain, which yields improved contrast, enabled visualisation of shadow nuclei. As the decomposition proceeded, the shadow nuclei were reduced in number. The stages of the cell nuclei are illustrated in Fig. 3.

The cytoplasm of hepatocytes displayed an apparent heterogeneity throughout the dataset, as granules and vacuoles were present in the majority of specimens, regardless of the PMI. The cytoplasm could appear well-stained with a dense appearance. In some cases, it was the opposite, with a poorly stained, clear cytoplasm (e.g. lacking glycogen). The appearance of cytoplasm was not scored in the final HDS system. Instead, the focus was on the cell structure of the hepatocytes, the degree of disintegration, and the increasing presence of cell debris (Fig. 3). The hepatocytes detached from the hepatic cords and became scattered, surrounded by cell debris. This detachment started early, at 1 to 5 days after death (Table 2), and was extensive between 10 and 20 days after death (Table 2). Thereafter, loss of cell material and tissue liquefaction were visible (Fig. 3e), in most cases after PMIs of 20 days (Table 2).

**Table 2:** The hepatic decomposition score (HDS) system (final version). The HDS system is based on examinations of conventional histological staining (HE, PM) and a magnification of × 100, × 200, and/or × 400

Score	Hepatocytes		Portal areas		Architecture
	Cell nuclei	Cell structure	Bile ducts	Portal triad	Micro and lobular
0	Distinct	Maintained	> 75% distinct nuclei (possible detachment from bile duct wall)	Distinct	Distinct lobules Distinct alignment of hepatocytes Distinct sinusoids
1	Darker, dense	Mild disintegration Signs of cell debris	> 25% distinct nuclei	Mild disintegration Indistinct borders Signs of collagen dispersion	Distinguishable lobules Mild loss of alignment of hepatocytes Distinguishable sinusoids
2	Shadows	Moderate disintegration Visible cell debris	Faded nuclei (solitary nuclei may be distinct)	Moderate disintegration Triad still distinguishable Collagen dispersion	Solitary distinguishable lobules may exist Moderate loss of hexagonal shape and alignment of hepatocytes Possible distinguishable sinusoids
3	Shadows sparsely located	Extensive disintegration Visible cell debris and loss of cell material	Cell residues (solitary nuclei may be distinguishable)	Extensive disintegration Blood vessels or bile ducts may still be distinguishable Collagen dispersion and signs of fragmentation	Blurred hepatic pattern Severe loss of alignment of hepatocytes and hexagonal shape Not identifiable sinusoids
4	Not identifiable	Complete disintegration Only some cell debris and collagen remain	Not identifiable as bile ducts	Complete disintegration Only collagen remains Collagen dispersion and fragmentation	Extensive blurred hepatic pattern Complete loss of alignment of hepatocytes Not identifiable sinusoids
5		Not identifiable Only collagen remains		Not identifiable as portal triad	Not identifiable No hepatic pattern remains

**Fig. 4:**
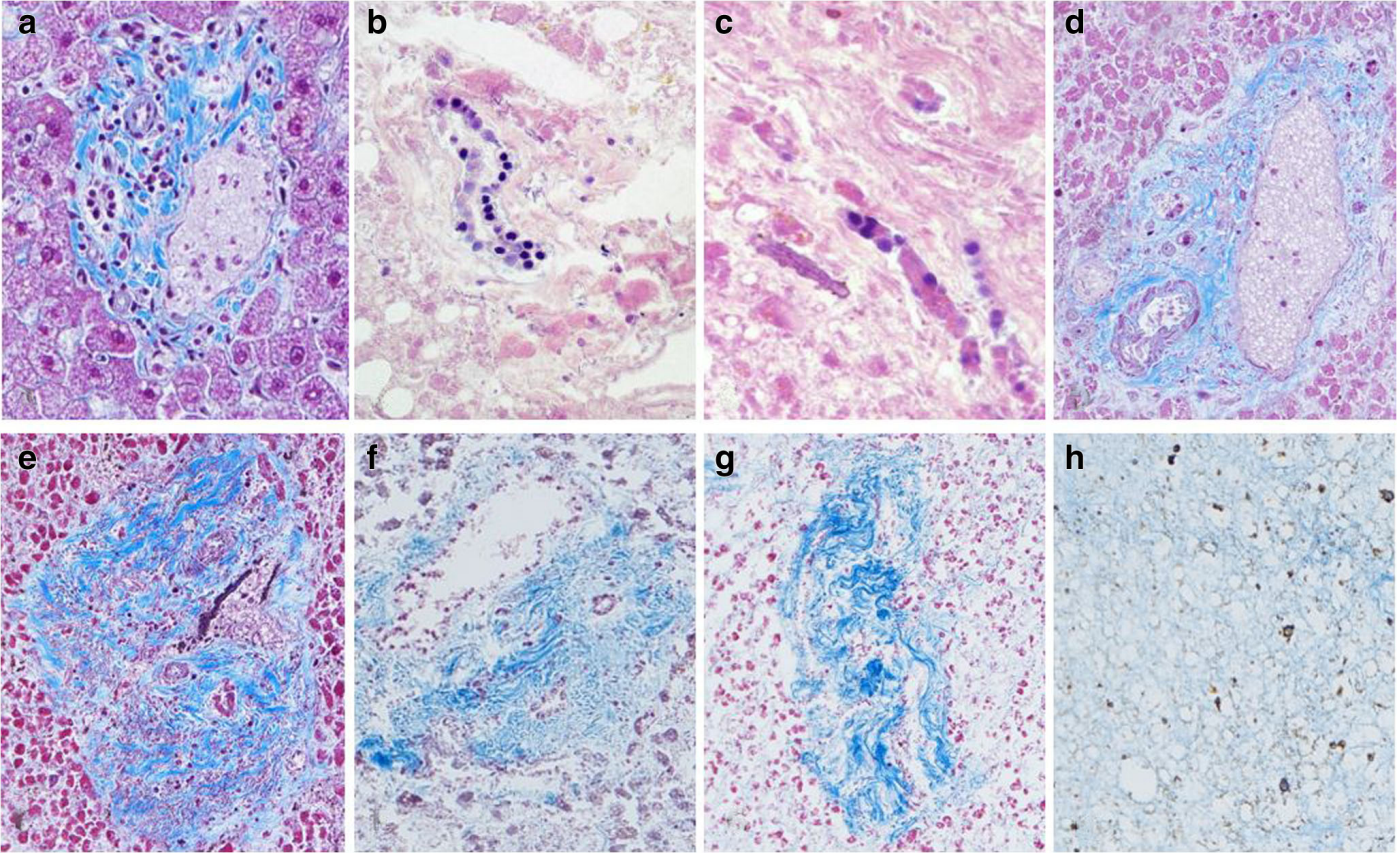
Decomposition of the portal area. **a** illustrates bile duct score 0 and portal triad score 0. More than 75% of the epithelial cell nuclei in the bile ducts are distinct. Stroma cells and leukocytes are possible to differentiate. All three components (artery, vein, and bile duct) of the portal triad are distinct. PM (Picro-Mallory trichrome) × 200. **b** illustrates bile duct score 1, with between 25 and 75% of the epithelial cell nuclei distinct. Some faded nuclei and cells without nuclei are also seen. HE (haematoxylin and eosin) × 100. **c** illustrates bile duct score 2. The epithelial cell nuclei are indistinct and/or faded. Cells without nuclei are also seen. HE × 200. **d** illustrates portal triad score 1; mild disintegration and early signs of collagen dispersion. All three components of the portal triad are still identifiable. In the PM stain, the collagen loses the dark blue colour and becomes lighter as the decomposition progresses. PM × 100. **e** illustrates portal triad score 2; moderate disintegration with collagen dispersion and signs of fragmentation. Bile duct score 3, with only cell residues remaining. PM × 100. **f** illustrates portal triad 3; extensive disintegration. Bile ducts or blood vessels may still be distinguishable. PM × 100. **g** illustrates portal triad score 4; complete disintegration, only collagen left, bile ducts and blood vessel not possible to identify with certainty. PM × 100. **h** illustrates portal triad score 5 (as well as cell structure score 5 and architecture score 5). Tissue no longer possible to identify as liver. Only remnants of collagen and brownish deposits visible

Prominent peripheral portal areas containing large blood vessels and bile ducts lined with columnar epithelium embedded in collagen were often well-preserved, decomposing at a slow rate compared with other liver structures. However, since they were not always present in the samples, they were discarded from the HDS system. Instead, the focus was on the portal triad (i.e. small blood vessels and bile ducts lined by cuboidal epithelium). In PM stain, the dark blue collagen progressively turned lighter as it slowly disintegrated (Fig. 4). The three components of the portal triad (i.e. artery, vein, and bile duct) were distinct during the first days after death, but started to disintegrate slowly. The blood vessels, as well as the bile ducts, were lost. The artery was more often resistant and could remain for a longer time period than the bile ducts. However, after a PMI of ≥ 20 days, it was difficult to distinguish blood vessels or bile ducts. Later, after a PMI of 30 days, they were no longer possible to identify with certainty. Cell debris could still be visible in the portal area, but was not easily identifiable as remnants from cell constituents of the blood vessels or bile ducts (Table 2). The cuboidal epithelium of bile ducts exhibited an optical disintegration with fading of the dark nuclei, and further formation of cell residues (Fig. 4). In advanced stages of decomposition, bile ducts were no longer distinguishable.

One specific feature that was discussed was the presence of bacterial formation of gas, but this feature was excluded from the HDS system as it was recognised as being independent of the PMI. Approximately 30% of all forensic autopsy cases displayed optical signs of gas formation of various sizes.

### The hepatic decomposition score system

The scoring system constructed consisted of five partial liver scores (HDS markers): *cell nuclei* and *cell structure* of the hepatocytes, *bile ducts*, *portal triad*, and *architecture*. The HDS markers could be used to calculate a total HDS (in analogy to TBS). For each individual marker, five or six stages of decay progression were defined (see Table 2). A score of 0 would imply no visible decomposition, a score of 1 would imply the lowest degree of decomposition, and a score of 4 or 5 (depending on the HDS marker) would imply the highest degree of decomposition. Each HDS marker was scored individually due to the different substructures decomposing at different paces. Differences in the degree of decomposition of a specific substructure within a specimen were treated as separate scores, with an average being calculated. During scoring, the entire liver specimen was examined initially. However, in samples with gas formation or fatty changes partly affecting the liver structure, at least five fields of view with assessable areas were needed to score the sample with certainty. The stages and description of HDS system are presented in Table 2.

### Testing the reliability of the HDS system

All the cases in dataset 2 (*n* = 154) were assessed blindly (i.e. without knowledge of PMI or TBS) by one observer (ASC). Of these cases, a sample of 40 was randomly selected for a secondary blinded assessment by another observer (HS), as well as an independent assessment by three observers without prior knowledge of the HDS system. During the pilot scoring, three of the authors (ASC, HS, SN) separately scored 31 of the 82 original cases (dataset 1) with a good (> 0.60) to excellent (> 0.75) ICC inter-observer agreement (Table 3). However, these liver slides had been assessed several times during the development of the HDS system. The HDS system was slightly modified after calibration discussions, during which specific cases were reviewed. When the cases were scored again, there may have been some bias. The new cases (dataset 2) were scored on only one occasion and results were not further discussed between the observers. The authors (ASC, HS) scored the sample of new cases in dataset 2 with excellent ICC inter-observer agreement, somewhat higher internally than with the independent observers (Table 3). A low SEM signifies a high level of score accuracy. In this study, the lowest SEM was reached in the final scoring as compared with both the pilot scoring and the independent observer scoring (Table 3).

**Table 3:** Intra-class correlation (ICC) and standard error of measurement (SEM)

	Cell nuclei ICC (SEM)	Cell structure ICC (SEM)	Bile ducts ICC (SEM)	Portal triad ICC (SEM)	Architecture ICC (SEM)
Pilot scoring	0.67 (0.73)	0.89 (0.45)	0.84 (0.53)	0.76 (0.53)	0.91/0.88* (0.31/0.34*)
Final scoring	0.87 (0.50)	0.91 (0.46)	0.94 (0.37)	0.94 (0.37)	0.94 (0.39)
Independent	0.62 (0.82)	0.79 (0.52)	0.85 (0.51)	0.85 (0.51)	0.78 (0.63)

*In the pilot scoring, the architecture was subdivided into “micro” and “macro”, while in the finalised version these two had been merged into a single HDS marker. After the final scoring, the decision was made to change the scale and start from 0 (zero) instead of 1 (one)

### Transformation of HDS

**Fig. 5:**
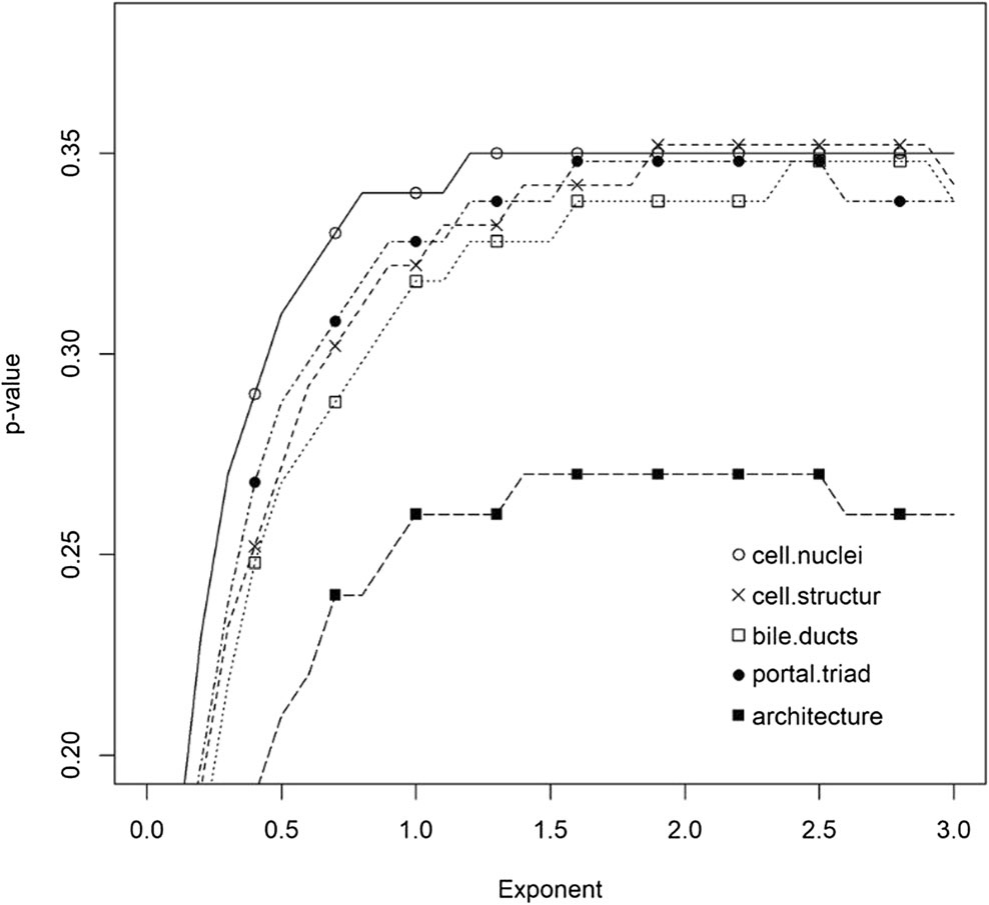
Testing different exponents and their *p* values. Values of the exponents, from 0 to 3, were tested in steps of 0.1. For each score, the model with the best *p* value was selected

**Fig. 6:**
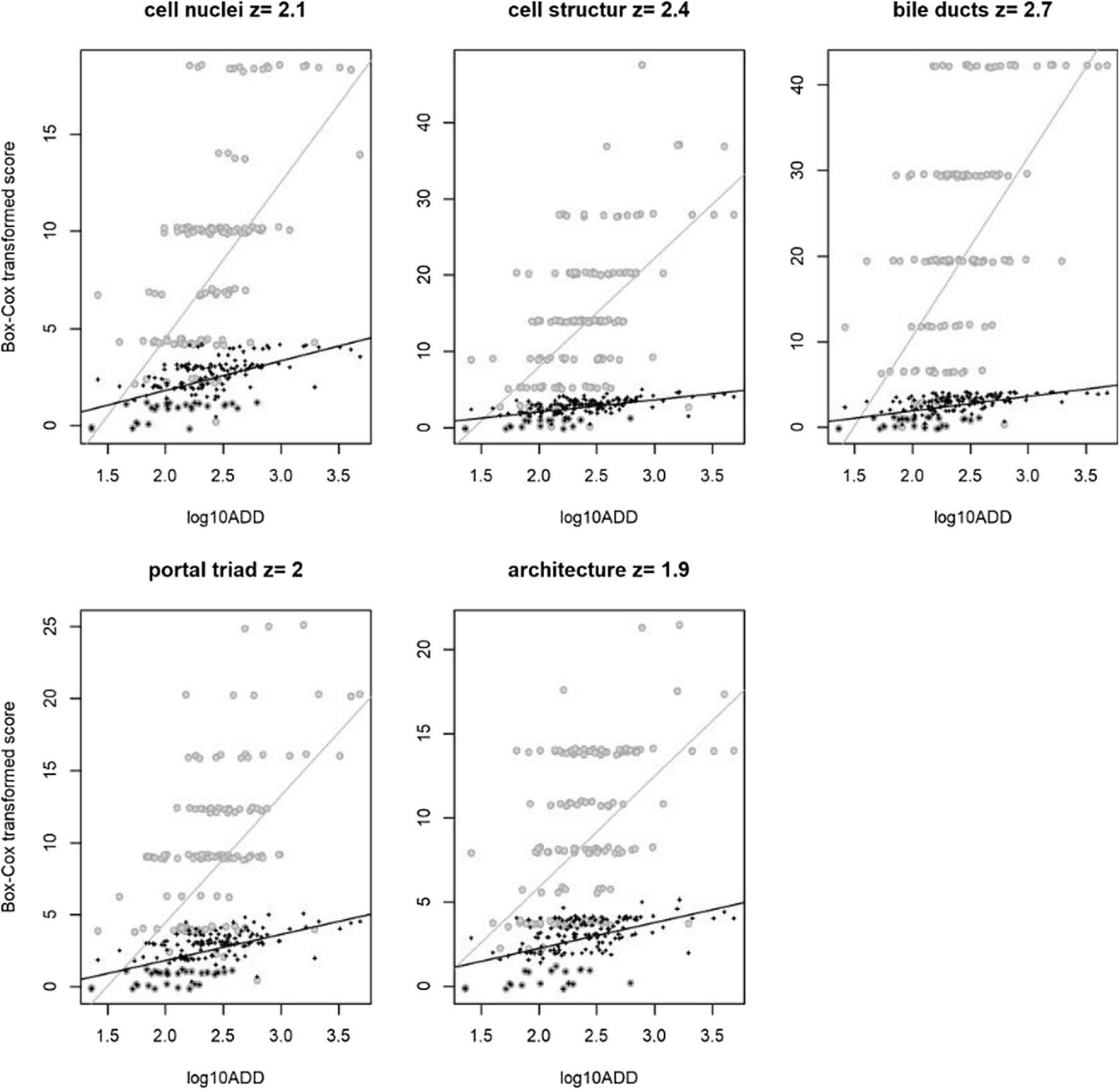
Linear regression. Log_10_ADD plotted against the untransformed (black) and the Box-Cox transformed HDS markers (grey). The optimal exponent for cell nuclei was 2.1; for cell structure, 2.4; for bile ducts, 2.7; for portal triad, 2.0; and for architecture, 1.9

In order to obtain the optimal fit for the model, different exponents for Box-Cox transformation were tested for the five HDS markers (*cell nuclei* and *cell structure* of the hepatocyte, *bile ducts*, *portal triad*, and *architecture*) in the training data. Values of exponents, from 0 to 3, were tested in steps of 0.1. For each score, the model with the best *p* value (i.e. when the model best explained the observed data) was selected (Fig. 5). Log_10_ADD was plotted against the untransformed and the Box-Cox transformed HDS markers, as illustrated in Fig. 6.

### Fitting and validation of the models

**Fig. 7:**
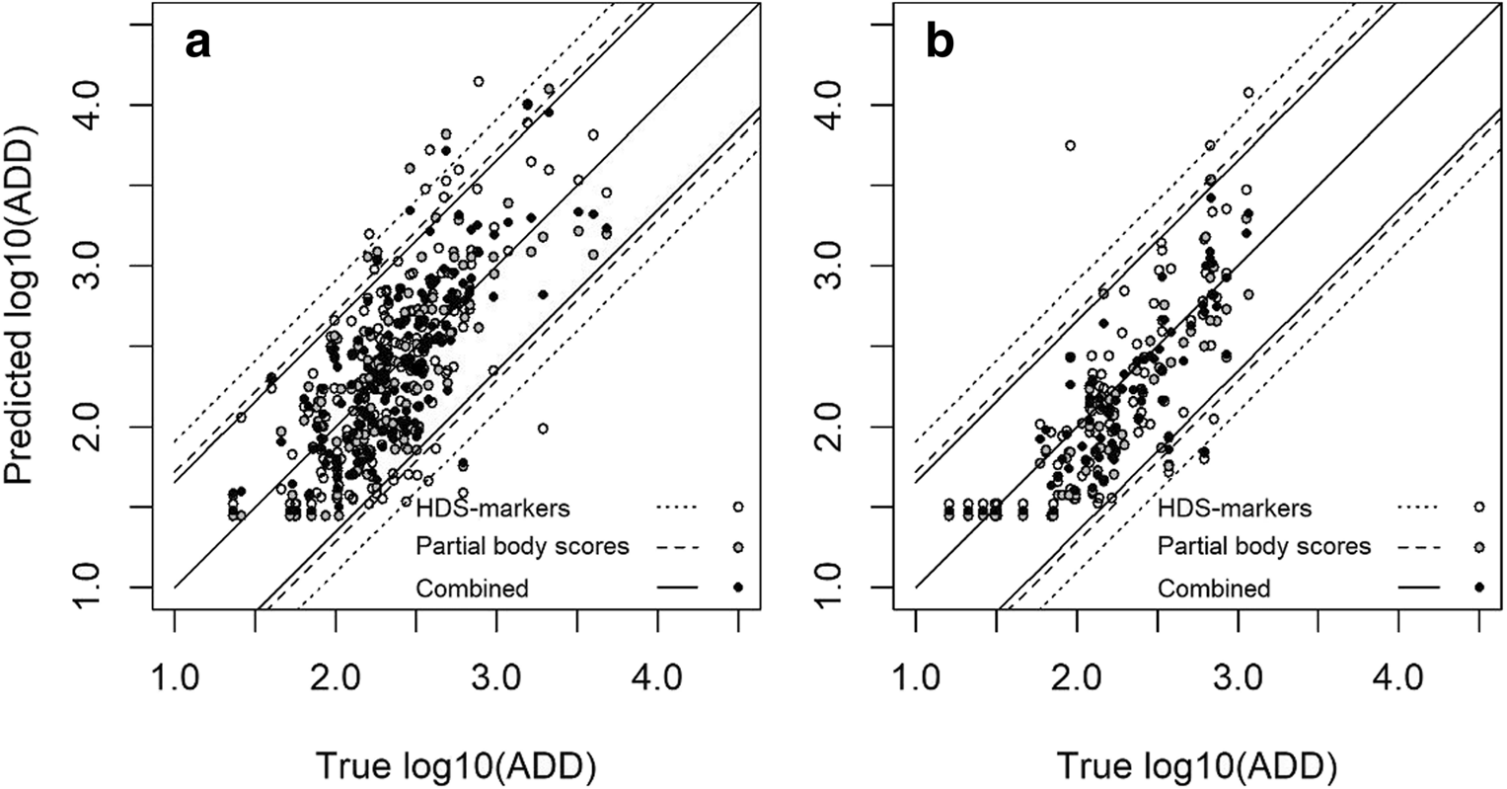
**a** Training data. **b** Validation data. The points indicate individual cases analysed with the model based on the five HDS markers only, the three partial body scores only, or all eight scores in combination. The *Y* axis indicates the value of Log_10_ADD that results in the highest likelihood for the observed HDS markers and partial body scores. The solid line indicates “perfect agreement”. The dashed lines are two standard deviations (according to the model), and we would expect that 95% of the samples would have a most likely ADD within this interval

The maximum likelihood method was used in training the models. After that, the fitted models were applied in order to calculate the value of log_10_ADD with the highest likelihood (i.e. at what ADD the scores would most likely be observed) in the training dataset. The true value of log10ADD was plotted against the estimated value (Fig. 7a). After this, the fitted model was applied to the validation data (Fig. 7b).

We tested how many of the predicted values of log_10_ADD (i.e. the value with the highest likelihood) were within the 95% confidence interval specified by the actual or true log_10_ADD for each individual case and the standard deviation (sigma) from the model. The highest sigma of 0.454 was seen for the model with the HDS markers. The sigma for the model based on only partial body scores was 0.358. A slight improvement was seen in the model when combining HDS markers and partial body scores, yielding a sigma of 0.328. In the training dataset and the validation dataset, the numbers of cases found within the confidence intervals of the three models were rather similar. The model with HDS markers had 95% of cases within the interval (representing two SD) for both the training dataset and the validation dataset. The model with partial body scored 96%, and the combined model scored 96% for training data and 97% for validation data.

The following coefficients were estimated in the linear regression model (presented in Table 4). Note that the Box-Cox transformed HDS markers had higher values on average, and a steeper slope, than the untransformed partial body scores.

**Table 4:** Coefficients and slopes in the linear regression model. HDS markers: cell nuclei (Nucl), cell structure (Struct), bile ducts (Bile), portal triad (Portal), and architecture (Arch). Partial body scores: head (PBSH), trunk (PBST), and limbs (PBSL)

	Nucl	Struct	Bile	Portal	Arch	PBSH	PBST	PBSL
*a* = slope	8.02	14.22	20.92	8.82	6.59	3.37	2.42	2.49
*b* = intercept	− 11.52	− 20.46	− 31.15	− 13.15	− 7.31	− 4.96	− 3.28	− 3.72

### Covariance

We investigated the covariance in the multivariate regression model applied to the datasets. Table 5 shows the correlation matrix, i.e. the covariance matrix divided by the variance of each HDS marker or partial body score. This gives an indication of the variance relative to the absolute value of the HDS marker or partial body score, or how dependent the different HDS markers and partial body scores are of each other. The HDS markers appeared to have slightly higher variance than the partial body scores. However, it could also be noted that the HDS markers appeared to have a low covariance with the partial body scores. Within the set of HDS markers and the set of partial body scores, respectively, the covariance was relatively high.

**Table 5:** The correlation matrix from the multivariate regression model. HDS markers: cell nuclei (Nucl), cell structure (Struct), bile ducts (Bile), portal triad (Portal), and architecture (Arch). Partial body scores: head (PBSH), trunk (PBST), and limbs (PBSL)

	Nucl	Struct	Bile	Portal	Arch	PBSH	PBST	PBSL
Nucl	1	0.67	0.59	0.6	0.6	0.22	0.24	0.37
Struct	0.67	1	0.49	0.6	0.86	0.22	0.14	0.31
Bile	0.59	0.49	1	0.81	0.49	0.43	0.38	0.49
Portal	0.6	0.6	0.81	1	0.58	0.45	0.37	0.47
Arch	0.6	0.86	0.49	0.58	1	0.24	0.16	0.33
PBSH	0.22	0.22	0.43	0.45	0.24	1	0.62	0.64
PBST	0.24	0.14	0.38	0.37	0.16	0.62	1	0.53
PBSL	0.37	0.31	0.49	0.47	0.33	0.64	0.53	1

### Performance when one or more scores were missing

We tested if the model’s performance and precision were affected if one or more HDS markers or partial body scores were missing. In order to determine this, test cases were predicted, with HDS markers or partial body scores replaced by N/A. As seen in Table S1 (supplement), different combinations of missing HDS markers and/or partial body scores were tested. Excluding a single HDS marker had little impact on the model. Excluding all HDS markers had approximately the same effect on the model as only excluding the partial body score of limbs (PBSL). If both PBSL and all HDS markers were missing (the body only has the head and trunk available for scoring), this had a greater impact on sigma and the model lost some of its precision. Excluding PBSL resulted in a sigma of 0.35, and excluding five HDS markers plus PBSL yielded a sigma of 0.40. When all HDS markers and partial body scores were included in the model, the sigma was 0.22 (see further details in supplement, Table S1).

### Filtering data and outliers

Having just a few outliers may have a large impact on the estimated sigma (SD) and consequently on the precision of the model. When we applied the 82 original cases, used to develop the HDS system, as training data for the statistical model, the model based on only HDS markers performed better than the model based only on partial body score (results not shown). To test the impact of outliers on model precision, we identified the cases where the predicted ADD was furthest from the actual true ADD in the models based on partial body scores and HDS markers, respectively. The distance was measured as Cook’s distance (Table S2, supplement). Creating a model where these cases were excluded resulted in significantly narrower intervals and smaller sigma.

In the training data, only a few cases had a large Cook’s distance in the regression model for true ADD and predicted ADD using HDS markers or partial body scores (Table S2, supplement). One case had a PMI of 86 days and a BMI of 15 kg/m^2^, with the body presenting external desiccation. Cook’s distance for HDS and partial body scores was 0.19 and < 0.01, respectively. For the other cases in the training dataset with PMIs of > 70 days, Cook’s distance for partial body scores ranged from 0.08 to 0.11, but little or no effect was seen on Cook’s distance for HDS markers in the same cases. The case characteristics were external desiccation and a relatively low BMI (i.e. 11 to 15 kg/m^2^). In the validation dataset, it was also seen that cases with longer PMIs (> 27 days) stood out with greater Cook’s distances, regarding both HDS and partial body scores. External desiccation or low BMI was not as evident in those cases as in the training dataset. However, one extreme case with Cook’s distance of 0.17 for HDS, but < 0.01 for partial body score, had a PMI of only 3 days, but a liver tissue in advanced decomposition.

When comparing the BMI in the outliers and the non-outliers, there was no evident association between low BMI and being an outlier (results not shown). Still, cases with extremely low BMI may be a potential source of outliers.

### Presence of desiccation

We also investigated if the presence of desiccation affected model precision. Of the 236 cases, approximately 34% displayed desiccation to some extent. Evaluating only desiccated cases seemed to improve model precision (i.e. smaller sigma) in comparison with cases without desiccation (representing cases with moist decomposition), but there were several extreme outliers, especially concerning partial body scores in cases without desiccation. There was a low number of cases with extensive soft tissue loss as the result of insect infestations. The significance of the comparison of model performance in the presence or absence of desiccation is questionable. The dataset of desiccated cases had a significantly higher proportion of extended ADD. This probably reflects the fact that the data contained no information on whether a case with low ADD had progressed under conditions that would favour desiccation or moist decomposition. In addition, when applying the Box-Cox transformation of the HDS markers, the optimal exponent was found to differ between cases with or without desiccation (results not shown).

## Discussion

The novel HDS system is indicated to be a statistically robust way of quantifying the degree of decomposition, as well as a useful aid in future PMI estimation. While our model has limitations, including a potentially large uncertainty in some cases (i.e. a low precision), the HDS system represents an important step towards a practical model for PMI estimation in forensic casework. As suggested by Madea et al. [23], even PMI methods with low precision can be useful in forensic casework. The novel scoring-based method also offers a structural and systemic evaluation of the decomposition process, the effects of different intrinsic and extrinsic factors, and their possible correlation with PMI.

### The decomposition of the human liver

Previous research on the sequential decomposition of the human liver is scarce and focused only on structural changes occurring in the first days after death [10–12], making our HDS method unique in this context. It is therefore difficult to compare our results with other publications. Kushwaha et al. [10] presented a scoring system where decomposition changes were graded on a scale 0 to 4, based on the appearance of portal areas, hepatocytes, and the liver architecture, observed at PMIs of 7–34 h. Structural changes, such as declining optical quality of the portal triad and disturbance of the lobular architecture, were similar to findings in our study. Verma et al. [12] focused on the architecture of hepatic lobules and the alignment of hepatocytes at PMIs up to 46 h. There, the hepatic lobules were described as disorganised and hepatocytes were completely separated from each other. These changes seemed to start evolving 13 to 14 h after death. Similar changes were also observed in our autopsy cases, but were generally noticeable slightly later. The majority of the cases with a PMI of less than 1 day in our study did not display any histological changes when examined under a light microscope. Usually, histological changes were observed after a PMI of 1 day and were manifest after a PMI of 5 days. The study by Karadžić et al. [11] of the human liver’s ultrastructure using electron microscopy indicated that autolytic changes were visible already at 6 h after death. This indicates that the autolysis starts very early after death. We could not, with our methods, observe these very early signs of autolysis (e.g. define specific and PMI-dependent changes in the liver tissue possible to quantify). We saw presence of bacteria scattered in many of the liver tissue sampled, but only a few cases with multiple large colonies of bacteria within the liver tissue or in blood vessels. We also saw indirect signs of bacterial activity, i.e. rounded voids of various sizes interpreted as gas formation. Both bacteria and gas formation can be observed in both liver tissue samples without any other histological changes (and with a PMI of less than 1 day) and those with extensive decomposition changes. We may assume that the liver’s position in the abdominal cavity, in close proximity to the gallbladder and the pancreas (autolytic enzymes) as well as the intestinal tract (large bacterial load), would promote a relative rapid rate of decomposition in the liver, making liver tissue samples unusable in longer PMIs. However, the end stage (only collagen remains) is not reached until approximately 35 days after death. Several cases with extended PMIs (43 to 217 days) had not reached the end stage.

It was evident that a rise in ambient temperature was followed by an increased rate of liver autolysis [10–12]. Autolysis has a delayed onset and a slow progression rate in a cold environment. However, longer storage time at a morgue (i.e. 8 days or more) could result in severe autolytic alterations [24]. In our dataset, the median morgue time was 4 days and very few cases were stored for longer than 8 days. Presumably, the cold environment in the morgue has a modifying effect on the decomposition process, but we cannot determine to what extent this occurred in this present study. When calculating ADD, we took into account the time that the dead body spent in the morgue, as well as the morgue temperature. We do not know at what temperature the decomposition progress ceases [8]. Vass et al. [25] stated that decomposition occurred down to 0 °C and Micozzi [26] stated that no decomposition took place at temperatures lower than 4 °C. Questions have been raised about the accuracy of ADD models [27]. However, applying an ADD model gave us the opportunity to take temperature variation into account, as well as incorporating the time after death.

### Performance, reliability, and validity of the HDS system

In spite of the continuous nature of decomposition, a scoring-based method gives a good opportunity to describe and quantify the degree of decomposition in a concrete case. This also enables comparison between cases. There is a certain inherent degree of subjectivity in the scoring. Still, a structural way of assessing the decomposition in human remains would be of value, in both forensic research and forensic practice.

When investigating the reliability of scoring liver slides using the HDS system, the results indicated a high reproducibility and consistent scoring. However, an inter-observer variability, in a single case, can lead to greater differences with regard to the real PMI. The results also indicated that training and calibration meetings could increase the reliability and accuracy. Generally, cell nuclei had less agreement between different observers than architecture and cell structure.

The Box-Cox transformation of TBS was used in Megyesi et al. [8] with the exponent 2 and in Moffatt et al. [18] with an exponent 1.6. We have previously tested the TBS model in an indoor setting and found that transformation was not necessary for TBS in our dataset [1]. However, it was not known if transformation of the HDS markers would be needed to improve the model fit (i.e. achieve a normally distributed response). Test results indicated that the optimal exponent in our training dataset was 1.9 to 2.7, depending on which HDS marker was investigated.

The fitted model was applied on the independent validation dataset with similar results as in the training dataset, indicating high accuracy. The model based on partial body scores had a somewhat higher precision than the model based on HDS markers. However, the model based on HDS markers combined with partial body scores performed slightly better than the model based on only partial body scores or that based only on HDS markers, suggesting that improved precision was achieved when combining the two methods. The likelihood curve gave a reasonable indication of the degree of uncertainty built into the model. Further, when testing the performance of the model if one or more of the HDS markers and/or partial body scores was missing or unreliable, the results indicated that the partial body score of limbs (PBSL) had the greatest impact on the model precision. If PBSL is not available, the HDS markers may still contribute to improving the precision of the model.

### Outliers and possible ways to improve the model

One way to improve the precision of the model might be the exclusion of extreme outliers. This could result in narrower intervals and smaller sigma values (SD), i.e. better predictions. However, this would require objective criteria for identification of these outliers or when it would be justified to exclude them. Specified exclusion criteria have to be practical and possible to apply in new cases. Decomposition is a complicated process, in which many different factors play a role. Identifying these factors and to what extent they affect indoor decomposition would be of great importance. In our dataset, the post-mortem BMI displayed large variation between cases. An extremely low post-mortem BMI might result in a potential outlier in the model. Still, we did not find an evident association between low BMI and being an outlier.

The rate of decomposition may be affected by desiccation. Our previous study of indoor decomposition found that the rate was slower; the PMI may therefore be underestimated in cases with desiccation [1]. When dividing the 236 cases into two groups—with or without presence of desiccation—the precision of the model seemed to improve regarding the desiccated cases. However, the results were somewhat ambiguous. There were several extreme outliers, as well as an apparent difference in ADD between the two groups. If many cases (outliers) are removed in order to get an improved precision, the models may no longer reflect reality.

### Strengths of the novel model

The TBS method was suggested to have a moderate to low precision in an indoor setting [1]. An alternative to only using the sum of the partial body scores, i.e. the TBS, was sought, and a multivariate regression model was set up based on the three separate, but correlated, partial body scores of the head, trunk, and limbs. The precision of this new model was suggested to be improved [22]. Further, the results indicated that the HDS markers were relatively independent of the partial body scores (as shown in Table 5), giving a better modelling of the decomposition process, with the potential of improving PMI estimates. Uneven decomposition in a specific case can create difficulties in how to score and calculate the TBS to optimally represent the degree of decomposition. Further, the TBS or partial body scores only reflect the external decomposition changes. Combination with HDS markers enables an improved representation of the decomposition that has taken part within the body. The fitted model of both scoring methods can be used even if one or more scores/markers are missing, for example, if one partial body score is considered unreliable due to injuries or extensive pathological lesions in the skin, etc.

## Conclusions

The histological changes observed throughout the trajectory of decomposition in human livers were demonstrated to be important in the quest to improve methods for estimating the PMI. As yet, relatively little weight had been placed on research regarding the indoor environment, especially in relation to extended PMIs. Our approach, combining HDS markers with partial body scores, allows a comparison of the degree of decomposition between bodies. It also provides new information concerning different factors affecting the rate and pattern of decomposition, such as the effect of temperature, clothing/coverings, intoxication, and bacterial load. Furthermore, the precision in prediction of ADD will increase if uncertainties in the input data are limited. Refining the model further may be possible by adjusting for factors affecting the rate of decomposition.

## Supplementary information

The following are supplementary data to this article:

Supplementary figures and tables

Dataset

ESM 1 (DOCX 72 kb)
