# Supplementary material for: Histological quantification of decomposed human livers: a potential aid for estimation of the post-mortem interval?
Source: Int J Legal Med. 2020 Nov 24;135(1):253–67. doi: 10.1007/s00414-020-02467-x (PMC7782410; doi:10.1007/s00414-020-02467-x)
Supplement: Supplementary file 1 — (DOCX 72 kb) [file 414_2020_2467_MOESM1_ESM.docx]

**Supplementary material for** “Histological quantification of decomposed human livers: a potential aid for estimation of the post-mortem interval?”


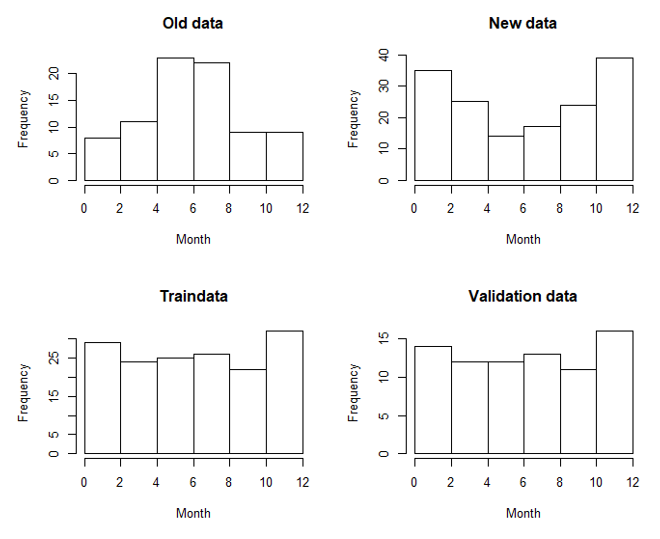


**Fig. S1.** Seasonal distribution within the old data (n = 82, original cases used in the development of HDS system, dataset 1) and the new data (dataset 2, n = 154). Based on the distribution of seasons in the two datasets, cases were sampled from the new data to obtain two datasets (i.e., the training data and the validation data) with approximately the same seasonal distribution.

| Included markers | |  | **Performance loss**  **Δ σ / σ_All_** | |
| --- | --- | --- | --- | --- |
| **HDS markers** | **Partial body score** | **µ** | **σ** | **(%)** |
| All | **All** | 2.65 | 0.22 | 0 |
| struct + bile + portal+ arch | **All** | 2.54 | 0.22 | 0 |
| nucl + bile + portal + arch | **All** | 2.69 | 0.22 | 0 |
| nucl + struct + portal + arch | **All** | 2.62 | 0.22 | 0 |
| nucl + struct + bile + arch | **All** | 2.64 | 0.22 | 0 |
| nucl + struct + bile + portal | **All** | 2.67 | 0.22 | 0 |
| All | **PBST + PBSL** | 2.65 | 0.22 | 0 |
| All | **PBSH + PBSL** | 2.66 | 0.23 | 5 |
| All | **PBSH + PBST** | 2.83 | 0.25 | 14 |
| - | **All** | 2.42 | 0.25 | 14 |
| All | **-** | 3.06 | 0.31 | 41 |
| struct + bile + portal + arch | **-** | 2.82 | 0.33 | 50 |
| nucl + bile + portal + arch | **-** | 3.19 | 0.32 | 45 |
| nucl + struct+ portal + arch | **-** | 3.01 | 0.33 | 50 |
| nucl + struct + bile + arch | **-** | 3.07 | 0.31 | 41 |
| nucl + struct + bile + portal | **-** | 3.09 | 0.31 | 41 |
| - | **PBST + PBSL** | 2.43 | 0.25 | 14 |
| - | **PBSH + PBSL** | 2.34 | 0.26 | 18 |
| - | **PBSH + PBST** | 2.51 | 0.31 | 41 |
| struct + bile + portal + arch | **PBSH+PBST** | 2.68 | 0.25 | 14 |
| nucl + bile + portal + arch | **PBSH+PBST** | 2.88 | 0.25 | 14 |
| nucl + struct + portal + arch | **PBSH+PBST** | 2.79 | 0.25 | 14 |
| nucl + struct + bile + arch | **PBSH+PBST** | 2.8 | 0.25 | 14 |
| nucl + struct + bile + portal | **PBSH+PBST** | 2.84 | 0.25 | 14 |
| All | **PBST + PBSL** | 2.89 | 0.26 | 18 |
| All | **PBSH** | 2.85 | 0.25 | 14 |

**Table S1.** Testing the model’s precision when one or more HDS markers and/or partial body scores were missing.

- HDS markers: hepatocyte cell nuclei (nucl) and cell structure (struct), bile ducts (bile), portal triad (portal), and architecture (arch).
- Partial body scores: head (PBSH), trunk (PBST), and limbs (PBSL).

| **Training dataset** | | | | **Validation dataset** | | | |
| --- | --- | --- | --- | --- | --- | --- | --- |
| HDS marker | | Partial body score | | HDS marker | | Partial body score | |
| *case ID* | *distance* | *case ID* | *distance* | *case ID* | *distance* | *case ID* | *distance* |
| **A09** | 0.02 | **A03** | 0.01 | **P03** | 0.02 | **P02** | 0.05 |
| **A18** | 0.02 | **A20** | 0.04 | **P07** | 0.04 | **P03** | 0.03 |
| **B33** | 0.02 | **B39** | 0.06 | **P11** | 0.05 | **P07** | 0.05 |
| **B39** | 0.02 | **B54** | 0.09 | **P34** | 0.01 | **P11** | 0.02 |
| **B53** | 0.07 | **C64** | 0.06 | **P49** | 0.01 | **P65** | 0.02 |
| **B54** | 0.04 | **C75** | 0.02 | **P56** | 0.17 | **P88** | 0.07 |
| **C64** | 0.03 | **C77** | 0.11 | **P57** | 0.16 | **P94** | 0.07 |
| **C72** | 0.03 | **C78** | 0.01 | **P64** | 0.01 | **P95** | 0.01 |
| **C75** | 0.01 | **C93** | 0.02 | **P84** | 0.02 | **P101** | 0.03 |
| **C78** | 0.02 | **C95** | 0.08 | **P88** | 0.02 | **P123** | 0.01 |
| **C95** | 0.01 | **C97** | 0.09 | **P93** | 0.01 | **P162** | 0.01 |
| **D132** | 0.01 | **C105** | 0.01 | **P101** | 0.03 | **P272** | 0.09 |
| **E144** | 0.02 | **D114** | 0.01 | **P129** | 0.02 | **P311** | 0.01 |
| **E148** | 0.01 | **D126** | 0.02 | **P272** | 0.05 | **P313** | 0.03 |
| **U047** | 0.02 | **D128** | 0.02 | **P313** | 0.02 | **U04** | 0.01 |
| **P12** | 0.01 | **D140** | 0.01 | **U05** | 0.01 | **U014** | 0.02 |
| **U060** | 0.19 | **E148** | 0.01 | **U014** | 0.07 | **U023** | 0.01 |
| **P116** | 0.01 | **P22** | 0.01 | **U024** | 0.02 | **U034** | 0.03 |
| **P92** | 0.05 | **P96** | 0.01 | **U028** | 0.08 | **U036** | 0.05 |
| **P05** | 0.05 | **P04** | 0.01 | **U032** | 0.02 | **U048** | 0.14 |
| **P125** | 0.01 | **P05** | 0.06 | **U048** | 0.09 | **U072** | 0.19 |
| **P130** | 0.02 | **U069** | 0.02 | **U072** | 0.01 |  |  |
|  |  | **P119** | 0.01 |  |  |  |  |

**Table S2.** The Cook’s distance. Testing the possible impact of outliers in the model’s precision. Cases were identified where the predicted ADD was most distant from the true ADD in the models, based on partial body scores and HDS markers, respectively. The table includes all cases where the Cook’s distance was > 0.01 in a regression for true ADD and predicted ADD. Left table (blue) shows the training dataset and the right table (green) shows the validation dataset.
